# From FGFR2 mutations to precision management: a review of prenatal diagnosis and multidisciplinary interventions in apert syndrome

**DOI:** 10.3389/fped.2025.1658654

**Published:** 2025-11-05

**Authors:** Hong Li, Junling Shen, Mei Tang, Siran Wan, Shunhong Zhang

**Affiliations:** ^1^ Department of Ultrasound Medicine, Panzhihua Women & Enfants Healthcare Hospital, Panzhihua, China; ^2^ Department of Gynaecology and Obstetrics, Yaan people’s Hospital, Yaan, China; ^3^ Department of Cardiology, Pangang Group General Hospital, Panzhihua, China

**Keywords:** apert syndrome, prenatal diagnosis, review, craniosynostosis, syndactyly, multidisciplinary management

## Abstract

Apert syndrome is a severe autosomal dominant disorder caused by recurrent FGFR2 mutations, characterized by the prenatal triad of craniosynostosis, midface hypoplasia, and symmetric syndactyly. This review synthesizes evidence defining core sonographic features: turribrachycephaly secondary to bicoronal suture fusion, facial profile abnormalities including depressed nasal bridge and hypertelorism, and the distinctive “mitten hands/sock feet” syndactyly pattern best visualized via advanced 3D ultrasound in late gestation. Fetal MRI complements ultrasound by identifying associated intracranial anomalies and microstructural brain changes linked to neurodevelopmental outcomes. A definitive diagnosis relies on targeted FGFR2 sequencing. Prenatal identification of these features enables essential coordinated care, including thorough parental counseling, proactive perinatal planning for potential airway compromise, and coordinated neonatal care involving craniofacial, genetic, and neurodevelopmental specialists. The integration of structured imaging assessment with rapid molecular diagnostics facilitates a shift from passive anomaly identification to proactive, risk-stratified management, thereby optimizing the long-term functional prognosis through timely interventions.

## 1 Introduction

Apert syndrome (OMIM #101200) is a severe autosomal dominant disorder caused by recurrent pathogenic variants in the FGFR2 gene, predominantly p.Ser252Trp and p.Pro253Arg in exon 7 (1, 2). Its clinical hallmarks—bicoronal craniosynostosis, midface hypoplasia, and symmetric complex syndactyly—form a recognizable triad that necessitates lifelong, multidisciplinary intervention (3). With an incidence ranging from 1 in 65,000 to 1 in 160,000 live births (4, 5), this condition exemplifies dysregulated craniofacial and limb development (3). Recent molecular investigations have revealed that the unregulated activation of FGFR2-driven ERK/MAPK signaling cascades accelerates premature osteogenic differentiation in cranial neural crest cells, thereby establishing a mechanistic link between the development of craniosynostosis and syndactyly (6, 7).

Prenatal diagnosis is pivotal for parental counseling, perinatal planning, and optimizing neurodevelopmental outcomes (8). While ultrasonography remains the primary screening tool, its sensitivity for detecting key features, particularly syndactyly, is variable (9, 10). This diagnostic inconsistency underscores the need for standardized imaging protocols. Current research focuses on enhancing detection through 3D HDlive Flow and spatiotemporal image correlation (STIC), which improve the visualization of syndactyly by reconstructing digital movement (11, 12). The integration of fetal MRI has emerged as a critical adjunct, particularly for evaluating associated intracranial anomalies. Studies utilizing diffusion tensor imaging (DTI) reveal white matter abnormalities in 68% of FGFR2-mutated fetuses, which correlate with postnatal neurocognitive impairment (13). Nevertheless, accessibility and cost constraints limit the routine use of MRI, reinforcing the frontline role of ultrasound.

Despite these advances, two persistent challenges hinder early diagnosis: (1) the phenotypic overlap with other FGFR-related craniosynostosis syndromes (e.g., Crouzon and Pfeiffer syndromes), which necessitates genetic confirmation; and (2) the inconsistent reporting of sonographic markers during mid-gestation, where facial dysmorphism may be subtle. This review synthesizes contemporary evidence to: (1) define evidence-based prenatal ultrasound criteria for Apert syndrome, incorporating recent advances in 3D imaging; (2) establish the complementary roles of MRI and FGFR2 sequencing within a stratified diagnostic pathway; (3) analyze the impact of early diagnosis on multidisciplinary care coordination, with an emphasis on perinatal airway management; and (4) discuss emerging molecular therapies (e.g., MEK inhibitors) transitioning from preclinical models to clinical trials. By addressing these objectives, we aim to provide a clinically actionable framework that bridges molecular insights with practical prenatal management.

## 2 Search strategy and selection criteria

A systematic literature search was conducted to identify relevant publications spanning from database inception to December 2024. The electronic bibliographic databases queried included PubMed/MEDLINE, Embase, Web of Science Core Collection, and the Cochrane Central Register of Controlled Trials. The search strategy was designed to encompass key concepts related to Apert syndrome, its molecular basis, prenatal diagnosis, and management. The following search terms and their variants were employed using Boolean operators: (“Apert syndrome” OR “Acrocephalosyndactyly”) AND (“FGFR2” OR “Fibroblast Growth Factor Receptor 2” OR “S252W” OR “P253R”) AND (“prenatal diagnosis” OR “prenatal ultrasound” OR “prenatal imaging” OR “fetal MRI” OR “magnetic resonance imaging”) AND (“craniosynostosis” OR “syndactyly” OR “mitten hand” OR “sock foot”) AND (“multidisciplinary management” OR “surgical intervention” OR “airway management” OR “EXIT” OR “neurodevelopment”).

The initial search results were screened by title and abstract to identify potentially eligible studies. Full-text articles were subsequently retrieved and assessed for final inclusion based on the following criteria: (1) original research articles, systematic reviews, meta-analyses, or seminal consensus guidelines; (2) publication in English; (3) primary focus on Apert syndrome or comparative analyses including Apert syndrome within FGFR-related craniosynostoses; (4) provision of substantive data or insights pertaining to prenatal sonographic/MRI features, molecular diagnostics, genotype-phenotype correlations, or multidisciplinary care pathways. Case reports were considered only if they presented novel diagnostic or therapeutic insights not covered in larger studies. References of included articles were also manually reviewed to identify additional pertinent publications not captured by the electronic search. The selection process prioritized recent high-impact studies from the past decade to reflect contemporary practice, while foundational historical papers were included for contextual completeness.

## 3 Characteristic prenatal ultrasound features: evidence-based criteria

### 3.1 Craniosynostosis: beyond turribrachycephaly

**Figure 1:**
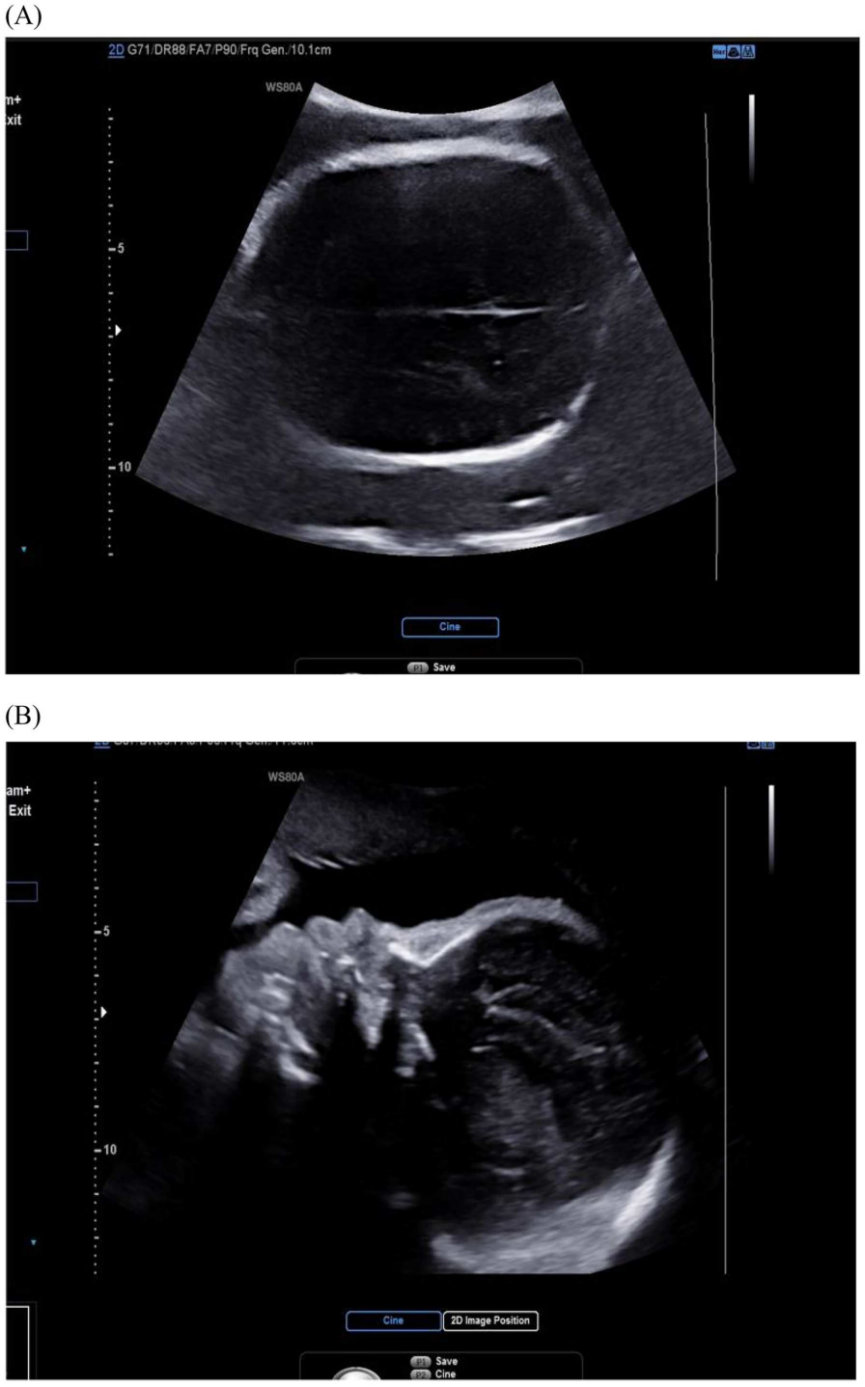
Ultrasound image of fetal skull in Apert syndrome: closed coronal suture (cross-section) and frontal bossing. **(A)** Cross-section view: the coronal suture, as seen in the cross-section of the skull, is closed and therefore not visible; **(B)** midsagittal view: the skull shape is abnormal, with a protruding forehead.

Bicoronal suture fusion constitutes the pathognomonic cranial hallmark of Apert syndrome, detectable sonographically from 18 gestational weeks. The most recognized manifestation is turribrachycephaly, characterized by a cranial index exceeding 90% (specificity 94%) and an abnormal fronto-occipital to biparietal diameter ratio >0.72, resulting in a towering cranial vault with frontal bossing (14). Crucially, modern ultrasound protocols now prioritize sutural biomarker analysis over isolated morphology assessment. Absence of the normal hypoechoic suture line, coupled with aberrant Doppler flow signals at coronal sutures, provides direct evidence of synostosis, reducing false positives from positional molding (10) (Figure 1).

Secondary intracranial signs further support diagnosis. The “copper-beaten” sign—inner table scalloping due to elevated intracranial pressure—is observed in 30% of cases after 26 weeks and correlates with postnatal ventriculomegaly risk (15). Recent technical advances enable earlier detection: high-frequency transvaginal probes (9–12 MHz) permit suture evaluation ≤16 weeks, identifying abnormal ossification centers in high-risk fetuses (16). This paradigm shift toward first-trimester risk stratification aligns with emerging evidence that cranial dysmorphogenesis initiates as early as 10–12 weeks in FGFR2-mutated embryos.

Three-dimensional ultrasound reconstruction with skeletal rendering mode (Classic, HDlive Silhouette) now quantifies suture fusion topography. Coronal synostosis typically extends 4–7 mm laterally from the sagittal midline, while associated lambdoid involvement manifests as posterior cranial flattening. Such precision facilitates differentiation from phenotypically overlapping syndromes like Saethre-Chotzen (unicoronal fusion) and Crouzon (multi-suture involvement without syndactyly) (9).

### 3.2 Midface hypoplasia: quantitative phenotyping revolution

**Figure 2:**
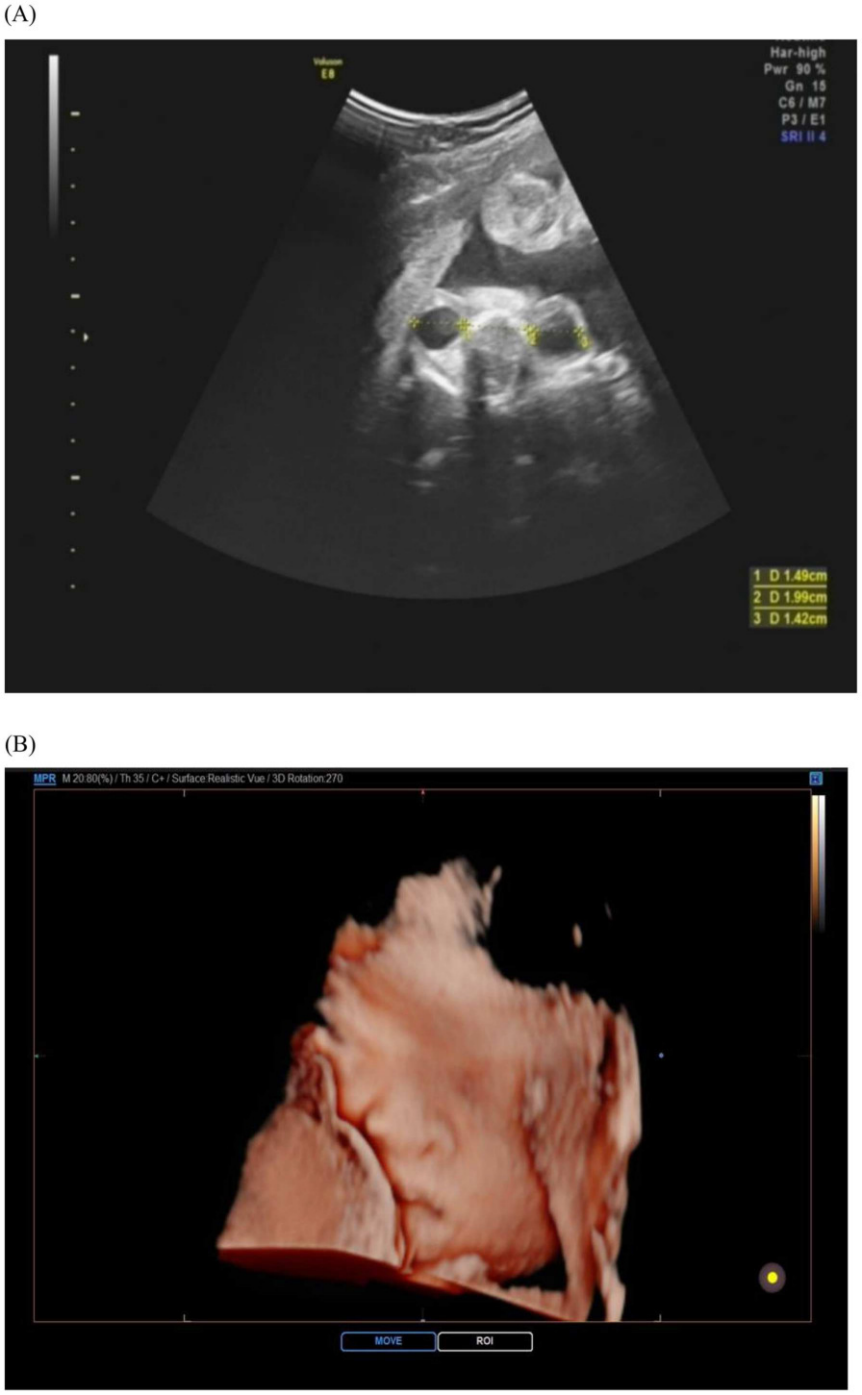
Ultrasound images of fetal face in apert syndrome. The cross-section of the bilateral orbits **(A)** and the three-dimensional view of the face **(B)** reveal a depressed nasal bridge and hypertelorism. The orbits appear shallow, resulting in relatively prominent eyes.

Midface retrusion in Apert syndrome represents a spectrum of deformities now quantifiable through standardized biometric approaches. Hypoplastic nasal bones, consistently reported as abnormally shortened in prenatal imaging, serve as a key diagnostic marker with high positive predictive value, outperforming subjective visual assessments (17). Concurrently, orbital hypertelorism (increased interorbital distance) and proptosis (anterior displacement of the globes) reflect impaired maxillary advancement, detectable via transorbital sonographic planes (18). The integration of three-dimensional angular phenotyping has refined diagnostic precision. A reduced maxilla-nasion-mandible angle demonstrates high specificity for severe midface deficiency and correlates strongly with postnatal airway compromise risk (19, 20). This shift to angular metrics aligns with developmental studies confirming FGFR2-mediated disruption of maxillary ossification centers as early as the first trimester. Computational modeling further validates that quantitative facial angle analysis provides earlier and more objective detection than traditional linear measurements (21) (Figure 2).

### 3.3 Syndactyly: high-definition dynamic imaging

**Figure 3:**
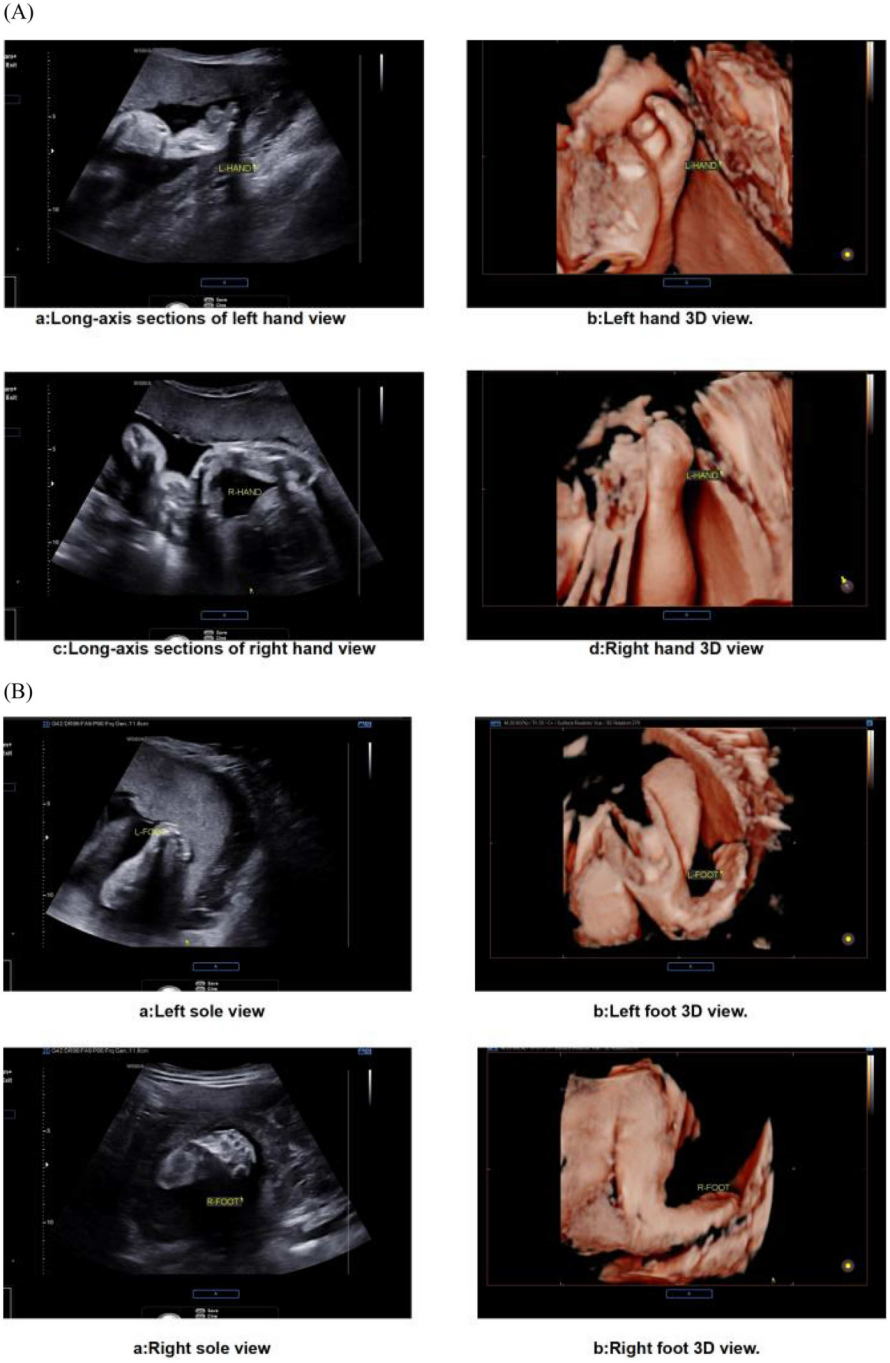
Ultrasound images of fetal extremities in apert syndrome. **(A)** The presence of an abnormal hand shape, a fixed posture, and an atypical arrangement of the metacarpal bones and phalanges is indicative of the “glove sign”. **(B)** The feet display a “glove sign”.

The prenatal assessment of syndactyly in Apert syndrome has evolved from static anatomical observation to dynamic functional evaluation (22). Traditional two-dimensional ultrasound primarily detects osseous fusion but exhibits limitations in visualizing cutaneous syndactyly due to technical constraints (23, 24). Advanced four-dimensional HDlive Flow imaging with spatiotemporal correlation overcomes these limitations by reconstructing perfusion patterns within fused digits (25). This technique discriminates Apert-specific “mitten hands” from other syndromic syndactylies by mapping unique vascular architectures, significantly improving diagnostic accuracy over conventional methods. Further refinement is achieved through reverse-mode rendering algorithms, which isolate osseous margins to reveal pathognomonic fourth metacarpal-phalangeal synostosis—a highly specific feature of Apert syndrome (26). Optimal visualization requires late-gestation imaging (>24 weeks), when digital ossification nears completion. Detection sensitivity progressively increases during this period, correlating with advancing skeletal maturation (27). Emerging artificial intelligence tools now automate digit segmentation, reducing operator-dependent variability in assessment (28, 29) (Figure 3).

## 4 Advanced imaging and genetic integration

### 4.1 Fetal MRI: beyond structural assessment

Advanced MRI transcends structural imaging to prognosticate neurodevelopment. T2 HASTE sequences confirm ventriculomegaly (≥10 mm) in 58% of cases and corpus callosum hypoplasia in 42%, yet these lack predictive value for cognitive outcomes (12, 30). Diffusion tensor imaging (DTI) addresses this gap: reduced fractional anisotropy in corticospinal tracts (68% of FGFR2-mutated fetuses) correlates with postnatal motor deficits (r = −0.71, *p* < 0.001), establishing prenatal connectomics as a biomarker (13). For cranial assessment, CISS 3D sequences map suture fusion topography, differentiating bicoronal synostosis (Apert) from unicoronal (Saethre-Chotzen) with 93% concordance to postnatal CT (31–33).

### 4.2 Molecular diagnosis: from karyotyping to next-generation paradigms

The diagnostic odyssey for Apert syndrome has evolved from chromosomal banding techniques to precision genomics. Conventional karyotyping, historically employed to exclude aneuploidies, fails to detect FGFR2 point mutations—a critical limitation given that >98% of cases stem from FGFR2 exon 7 variants (p.Ser252Trp or p.Pro253Arg) (34, 35). Sanger sequencing of this hotspot remains the diagnostic gold standard, achieving near-complete (99.7%) sensitivity in prenatal amniocytes when combined with multiplex ligation-dependent probe amplification (MLPA) to rule out mosaicism (36). Non-invasive prenatal testing (NIPT) using targeted digital PCR analysis of cell-free fetal DNA (cffDNA) demonstrates potential for detecting FGFR2 p.Ser252Trp variants, though current sensitivity remains suboptimal (<90%) in clinical practice due to biological and technical constraints. Fetal fraction thresholds exceeding 8% are required for reliable analysis, limiting its applicability in early gestation (37, 38). For equivocal cases, rapid whole-exome sequencing (rWES) of amniotic fluid delivers results within 7 days, resolving 92% of craniosynostosis syndromes with undetermined ultrasound findings (39). This acceleration is pivotal: prenatal diagnosis before 22 weeks optimizes counseling windows for termination decisions in jurisdictions with gestational limits. Emerging third-generation sequencing technologies promise further transformation. Oxford Nanopore long-read sequencing discriminates FGFR2 haplotypes at allele fractions as low as 0.1%, enabling non-invasive phasing of *de novo* mutations (40). CRISPR-Cas9-mediated enrichment of specific fetal alleles in maternal plasma demonstrates *in vitro* potential to enhance detection sensitivity (41). However, due to the limitations of fetal DNA content, it is not feasible to completely replace invasive testing in the short term.

## 5 Differential diagnosis

Other craniosynostosis syndromes, including Crouzon syndrome, Pfeiffer syndrome, and Saethre-Chotzen syndrome, also manifest with craniosynostosis; however, they exhibit distinct clinical and genetic characteristics. Crouzon syndrome is marked by craniosynostosis accompanied by normal limb development, whereas Pfeiffer syndrome may present with broad thumbs and toes (42). Saethre-Chotzen syndrome is associated with variable craniosynostosis and additional anomalies such as ptosis and ear abnormalities (43). Accurate differentiation of these syndromes often necessitates genetic testing. Isolated syndactyly, which occurs without craniosynostosis, represents a more prevalent congenital anomaly. It can be differentiated from Apert syndrome by the absence of cranial and other associated anomalies, and the pattern and severity of syndactyly may also vary (43) (Table 1).

**Table 1:** Differential diagnosis of Apert syndrome and related craniosynostosis syndromes.

Syndrome	Genetic basis	Craniosynostosis pattern	Facial features	Limb abnormalities	Key distinguishing features	Diagnostic confirmation
Apert Syndrome	FGFR2 mutations (98% S252W/P253R) (2)	Bicoronal fusion → Turribrachycephaly	• Severe midface hypoplasia • Hypertelorism • Beaked nose	• Complex syndactyly • “Mitten hands” • “Sock feet"	Symmetric bony syndactyly + corneal suture fusion	FGFR2 exon 7 sequencing (2)
Crouzon Syndrome	FGFR3 mutations (44)	Multisuture (coronal > sagittal)	• Midface hypoplasia • Proptosis • Normal intelligence	Normal limbs	Absence of syndactyly	FGFR3 mutation analysis (45)
Pfeiffer Syndrome	FGFR1/FGFR2 mutations (46)	Coronal + sagittal	• Midface deficiency • Shallow orbits • Hearing loss (47)	• Broad thumbs/great toes • Partial syndactyly	Type 2/3: elbow ankylosis + neurological impairment (48)	FGFR1/2 testing (48)
Saethre-Chotzen Syndrome	TWIST1 mutations (70%) (49)	Unilateral coronal → Plagiocephaly	• Facial asymmetry • Ptosis • Low-set ears	• Brachydactyly • Mild cutaneous syndactyly (50)	Ptosis + ear anomalies (50)	TWIST1 sequencing (49)
Muenke Syndrome	FGFR3 P250R mutation (51)	Unilateral/bicoronal	• Mild midface hypoplasia	• Carpal fusion • Conductive hearing loss (52)	Sensorineural hearing loss (35%) (52)	FGFR3 P250R specific test (51)
Isolated Syndactyly	Variable (HOXD13, etc.) (53, 54)	Absent	Normal	Cutaneous/bony fusion (variable digits) (55)	No craniofacial involvement (55)	Clinical + radiographic exam (53)

## 6 Critical implications of prenatal diagnosis

### 6.1 Parental counselling and multidisciplinary planning

Parental Counselling: Prenatal diagnosis allows the parents to decide on the continuation or termination of pregnancy. It also makes early psychological and social support possible. Parents can be counselled on the expected outcome, possible complications, and the need for multidisciplinary management after birth (56). Delivery Planning: Knowledge of the diagnosis enables the practitioner to plan for the mode of delivery. In cases where there is severe craniosynostosis or suspicion of airway obstruction, consideration for cesarean delivery may be entertained to minimize birth trauma and to ensure immediate access to neonatal resuscitation and management (57). Multidisciplinary Management Activation: Prenatal diagnosis creates a unique window to engage key specialists—neonatologists, craniofacial surgeons, geneticists, and neurodevelopmental therapists—before birth, facilitating pre-delivery care planning and role assignment. This early activation ensures that the care team is prepared to address immediate postnatal needs (e.g., airway support, initial imaging) and establish a long-term care roadmap, which is critical for optimizing the affected child's functional outcomes (58).

### 6.2 Summary of prenatal markers and clinical action pathway

**Figure 4:**
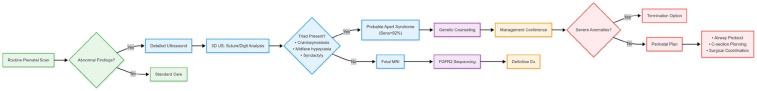
Apert risk stratification diagnostic flowchart.

Building upon these critical implications, the integration of key prenatal findings triggers a defined clinical action pathway. Prenatal suspicion arises from key ultrasound markers: turribrachycephaly, midface hypoplasia with hypertelorism, and symmetric “mitten hands/sock feet” syndactyly. Therefore, the identification of any of these features, particularly in combination, should not be viewed in isolation but should prompt a standardized diagnostic cascade. The immediate clinical actions following initial sonographic suspicion are twofold. First, a detailed, targeted ultrasound examination, preferentially employing 3D/4D modalities, should be performed to confirm and characterize the extent of anomalies. Second, this can be followed by a specialist fetal MRI to exclude associated structural brain anomalies and assess white matter microstructure, depending on clinical circumstances and resource availability. The definitive diagnostic step remains invasive testing via amniocentesis for targeted FGFR2 gene sequencing to confirm the molecular diagnosis. This sequential diagnostic approach—from ultrasound suspicion to molecular confirmation—ensures diagnostic rigor and immediately initiates the transition to coordinated care, including parental counseling, perinatal planning, and multidisciplinary postnatal management. To facilitate this integrated diagnostic and management approach, we propose a risk stratification diagnostic flowchart based on key sonographic findings and subsequent steps (Figure 4).

## 7 Lifespan management: critical phases from perinatal to childhood

### 7.1 Perinatal phase: airway-centric protocols

Given the high risk of airway obstruction in Apert syndrome secondary to severe midface hypoplasia and cranial base anomalies, pre-delivery mobilization of a dedicated Airway Multidisciplinary Assessment Team (AMAT) is essential (59). This team—integrating maternal-fetal medicine, neonatology, pediatric otolaryngology, and anesthesiology—systematically implements risk-stratified interventions: for critical cases, Ex Utero Intrapartum Treatment (EXIT) procedures with secured tracheostomy are prioritized to address immediate respiratory failure, utilizing real-time laryngoscopy to navigate anatomical complexities; for less severe phenotypes, nasopharyngeal intubation or Continuous Positive Airway Pressure (CPAP) support post-cesarean delivery proves effective (60). Consequently, the predominance of cesarean sections reflects proactive adaptation to turribrachycephalic cranial constraints, with this protocolized, team-based approach significantly mitigating neonatal mortality compared to historical *ad hoc* management (59).

### 7.2 Infancy (0–2 years): cranial expansion, neuroprotection, and early hand function

Early cranial vault remodeling constitutes the cornerstone of infant management in Apert syndrome, driven by the imperative to mitigate intracranial hypertension and its irreversible neurocognitive sequelae. Given the accelerated calvarial fusion kinetics, fronto-orbital advancement is prioritized within the first year of life (61), where surgical release of fused sutures not only expands intracranial volume but also reconfigures the orbital framework to protect globes from exposure (62). Contemporary management has evolved, with growing emphasis on primary posterior cranial vault distraction osteogenesis (PCVDO) for initial treatment. Research indicates that PCVDO safely achieves significant cranial volume expansion (approximately 20%–25%) and effectively controls intracranial pressure, serving as a robust intervention that can delay the need for complex fronto-orbital advancement (FOA) (63–66). Alternatively, minimally invasive strip craniectomies performed before 6 months of age, followed by helmet therapy, have established a key role in early management. Multicenter studies confirm that various minimally invasive techniques reliably correct the scaphocephalic deformity by harnessing the brain's rapid growth potential for physiological calvarial remodeling (67–70). It is important to note that infantile surgical management encompasses multiple options—including early PCVDO, early minimally invasive strip craniectomies, or later FOA—and may involve combined procedures at distinct timepoints (71). Together, these approaches represent a strategic shift towards initial, less invasive procedures that prioritize neuroprotection and growth modulation, potentially reducing the burden of major reconstructive surgery in early infancy.

Concurrently, functional restoration of the extremities begins in this period. The initial and most critical step in syndactyly management—the release of the thumb and first web space—is typically performed around 6 months of age. This early intervention is fundamental for establishing basic grasp, a milestone that unlocks essential sensorimotor development and enables the infant's active interaction with their environment (72). To preemptively address neurodevelopmental vulnerability, serial diffusion tensor imaging monitors white matter integrity, while structured enrichment programs targeting sensorimotor pathways are initiated upon detecting aberrant neural trajectories (30). This integrated paradigm—surgically optimizing physical containment while actively nurturing neural plasticity and establishing early hand function—establishes the foundation for functional outcomes beyond mere survival.

### 7.3 Childhood (3–12 years): functional reconstruction

**Figure 5:**
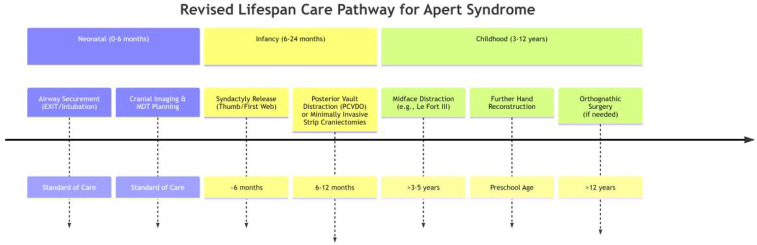
Lifespan care pathway for Apert syndrome: from neonatal to childhood interventions.

Functional restoration during childhood focuses on correcting midfacial retrusion and digit coalescence to enable essential breathing, vision, and manual dexterity, while concurrently addressing psychosocial barriers to social integration. The management of progressive midface hypoplasia becomes a primary focus during this stage. As progressive midface hypoplasia exacerbates obstructive sleep apnea and corneal exposure—potentially fueling social withdrawal due to visible facial differences—Le Fort III advancement with distraction osteogenesis is strategically timed prior to permanent dentition eruption, thereby simultaneously expanding the nasopharyngeal airway while repositioning the orbits for protective globe coverage and mitigating stigma-associated anxiety (73). Following the initial procedures in infancy, syndactyly reconstruction continues throughout childhood. Staged web space releases beyond the first web prioritize functional commissure formation over cosmetic outcomes, specifically enabling participation in peer activities (e.g., writing, play) critical for self-esteem development (74, 75). Furthermore, sensory integration protocols address high-frequency hearing loss through ventilatory tube placement to prevent academic disengagement from auditory processing deficits, and implement speech therapy to overcome articulation barriers from palatal dysmorphology, explicitly targeting communicative confidence in classroom settings (59, 76). This coordinated triad of skeletal, digital, and communicative interventions transforms passive anatomical correction into active participation in daily activities, with embedded cognitive-behavioral strategies reinforcing resilience against appearance-related bullying (77–79). The integrated care pathway across these critical phases is summarized in Figure 5.

## 8 Discussion and outlook

This review synthesizes pivotal advances in prenatal management of Apert syndrome, establishing that integrated imaging and molecular diagnostics are redefining prognostic precision. Crucially, the convergence of three-dimensional dynamic ultrasound, advanced fetal MRI, and rapid FGFR2 sequencing enables early risk stratification, transforming passive anomaly detection into proactive intervention planning. Beyond structural anomalies, emerging evidence links prenatal temporal lobe malformations and white matter microstructural alterations to neurocognitive outcomes, necessitating expanded counseling frameworks that incorporate neurodevelopmental prognostication and early enrichment strategies (77, 80). Furthermore, syndromic associations (e.g., shared RAS/MAPK dysregulation with neurocutaneous disorders) underscore the value of comprehensive phenotyping (81).

Nevertheless, despite significant advances in the prenatal diagnosis and management of Apert syndrome, several evidence gaps remain. First, the precise correlation between FGFR2 genotype and phenotypic severity is not fully elucidated, limiting accurate prognosis. Second, the predictive value of early (e.g., first-trimester) imaging biomarkers still requires validation in large prospective studies. Furthermore, the sensitivity of NIPT for detecting FGFR2 mutations needs improvement to potentially replace invasive diagnostic methods.

These unresolved questions are closely tied to the pathological mechanisms underlying Apert syndrome, which center on dysregulated FGFR2 signaling. Specifically, constitutive activation of FGFR2 drives abnormal bone differentiation primarily via the ERK/MAPK and p38 MAPK cascades (82), Meanwhile, the regulatory program mediated by Runx2/Sp7 integrates pathological effects in craniofacial and dental manifestations (83, 84). These insights illuminate therapeutic opportunities: Pathway modulators like MEK/p38 inhibitors show preclinical efficacy, though their application requires careful calibration to preserve physiological ossification (82, 85); conversely, CRISPR-based correction restores cellular function *in vitro*, yet *in vivo* delivery hurdles and oncogenic risks demand resolution before clinical adoption (86, 87). Importantly, the dual developmental-malignant roles of FGFR pathways necessitate rigorous safety frameworks.

In the realm of surgical management, a significant paradigm shift is underway, moving beyond the traditional emphasis on early fronto-orbital advancement. Growing evidence supports alternative initial strategies aimed at mitigating the high cumulative burden of multiple major craniofacial procedures. First, primary posterior vault distraction osteogenesis (PVDO) is increasingly advocated as the first intervention for several reasons: it provides a greater volumetric increase in intracranial capacity compared to anterior expansion, effectively addressing intracranial hypertension in infancy; it simultaneously ameliorates tonsillar herniation risks associated with turribrachycephaly; and by delaying complex fronto-orbital remodeling, it allows for further growth of the facial skeleton and maturation of the infant, potentially yielding more stable and favorable long-term aesthetic and functional outcomes (80). Second, the application of minimally invasive techniques, such as endoscopic strip craniectomy, offers a low-morbidity option for early suture release when performed before 6 months of age. This approach, coupled with postoperative helmeting, harnesses the rapid brain growth of infancy to drive calvarial remodeling, and may successfully delay or, in select cases, even circumvent the need for a formal anterior cranial vault expansion (81, 82). These evolving strategies underscore a more nuanced, individualized, and step-wise surgical philosophy that prioritizes neuroprotection and growth modulation while seeking to reduce overall morbidity.

Clinically, phenotypic heterogeneity—from subtle presentations to life-threatening airway compromise—demands stratified management. MRI volumetry objectively guides perinatal airway planning, supported by cost-benefit arguments for rapid genetic confirmation (88). Throughout the lifespan, staged reconstructive surgery should synchronize with neurocognitive support during infant plasticity windows, complemented by therapies addressing communicative barriers to optimize psychosocial outcomes (78).

Looking forward, transformative progress hinges on integrating several synergistic domains, including establishing international phenotyping standards, democratizing diagnostics via low-cost AI-ultrasound, exploring combinatorial pathway modulation, and developing global neurodevelopmental registries. To this end, a pivotal goal will be the creation of integrated AI platforms capable of synthesizing multi-modal phenotypic data—from 3D cranial vault shape and facial profile to digital morphology—into a unified diagnostic aid. Such tools would stratify the risk for syndromic craniosynostoses by analyzing the complete phenotypic triad, ultimately shifting management from reactive correction toward proactive prevention and precision diagnosis (89).

## 9 Conclusion

Prenatal diagnosis of Apert syndrome has transitioned from isolated anomaly detection to a molecularly integrated paradigm, where recognition of the sonographic triad is synergistically enhanced by fetal MRI biomarkers and rapid FGFR2 sequencing. This approach not only enables early risk stratification but also supports personalized intervention planning. Meanwhile, detailed mechanistic understanding of how FGFR2-mediated dysregulation of the p38 MAPK/Runx2-Sp7 axis drives disease pathology has opened up new horizons for therapeutic innovation, encompassing both pathway-modulating agents and CRISPR-based correction technologies. Critically, prenatal diagnosis transforms outcomes by facilitating proactive airway management, neuroprotective interventions, and staged surgical planning. Ultimately, the alignment of advanced diagnostics, targeted therapies, and specialty-specific care coordination—each tailored to the patient's developmental phase—promises to shift Apert syndrome management from reactive correction toward preventative precision medicine, optimizing functional autonomy across the lifespan.
